# Cost effective analysis after patient communication training in obstetrics - Evaluating economic efficiency

**DOI:** 10.1016/j.puhip.2025.100618

**Published:** 2025-05-13

**Authors:** Beate Hüner, Anand Kumar Vinayak, Martina Schmiedhofer, Christina Derksen, Frank Reister, Christoph Scholz, Sonia Lippke

**Affiliations:** a University Hospital Ulm, Department for Gynecology and Obstetrics, Prittwitzstr. 43, 89075, Ulm, Germany; b Constructor University Bremen, Campus Ring 1, 28759, Bremen, Germany; c Coalition for Patient Safety, Berlin, Germany; d Wolfson Institute of Population Health, Queen Mary University of London, Charterhouse Square, London, EC1M 6BQ, UK; e Munich Municipal Hospital Group, Department for Gynecology and Obstetrics, Germany; f Hamburg University of Applied Sciences/ Hochschule für Angewandte Wissenschaften Hamburg (HAW Hamburg), Ulmenliet 20, 21033 Hamburg, Germany

**Keywords:** Communication training, Adverse events, Patient safety, Obstetrics, Risk management, Cost effectiveness

## Abstract

**Background:**

In obstetrics, teamwork among healthcare professionals and effective communication with expectant parents are key to prevent adverse events during childbirth. These events can have lasting impacts on families and lead to significant costs for both the healthcare system and the affected families. The aim of this study is to evaluate the cost effectiveness of a training intervention for expectant mothers, focusing on improving effective communication in obstetrics.

**Study design and methods:**

An experimental intervention study was conducted with 76 pregnant women in the intervention group receiving patient training, and 88 in the control group. Cost effectiveness of the obstetric data was collected and evaluated with the Diagnosis Related Groups (DRG) classification, the internal cost of medical treatment and reimbursement by health insurance. In addition, patient characteristics, co-morbidities, and risk factors were assessed.

**Results:**

We found meaningfully lower costs per patient following patient training including communication training (patients with complications and in the intervention group 3053 Euros vs. with complications but in the control group 4523 Euros; patients without complications 2168 vs. 2418 Euros). The training significantly impacted patient safety in terms of a reduced average patient costs by 30 % only in case where women experienced complications.

**Conclusion:**

The results demonstrate the positive impact of communication training on enhancing patient safety while simultaneously reducing costs. The implementation of a patient training program focusing on communication skills for expectant parents effectively merges economic efficacy with a progressive approach in obstetrics. Furthermore, effective communication training could improve the articulation of needs, thereby bridging the gap between potentially exacerbated health inequalities for pregnant women with social risk factors.

## What this study adds

• Effective communication in obstetrics reduces preventable adverse events and healthcare costs.
• A patient training program that enhances communication skills effectively improves self-efficacy and patient satisfaction, which are vital components of an efficient and equitable healthcare system.
• Ineffective communication, particularly driven or exacerbated by social risk factors, plays a major role in healthcare inequalities. Therefore, implementing communication training is crucial for reducing disparities in care for expectant parents and improving obstetric care's efficiency and effectiveness.

## Implications for policy and practice

• Implementing communication training for healthcare workers and expectant mothers is crucial for empowering patients and optimizing care, aligning with policies that promote patient-centered practices and enhance patient involvement in healthcare decisions. This approach also improves patient safety by avoiding missed opportunities for support or healthcare services and minimizing miscommunication.
• Regular refresher courses on communication skills in clinical settings are essential for maintaining their effectiveness. Incorporating these into practice supports policies for continuous professional development and improves patient care and safety.
• It is crucial to explicitly address problems in accessing and receiving high-quality maternity care, which is especially challenging for parents from (ethnic) minorities. Culture-sensitive and equitable strategies to ensure patient-centered care and participation in research are needed.

## 1 Introduction

Efforts to improve patient safety are aimed at avoiding so-called preventable adverse events (pAEs). The definition of adverse events (AEs) and their root causes has been the subject of extensive scientific over the past decades [1]. Worldwide, an estimated 3–16 % of hospitalized patients experience erroneous medical treatment. This results not only in psychological and physical harm to the patient but also in an additional financial burden on the healthcare system [2,3]. According to the World Health Organization, the global annual economic impact of AEs is estimated to be between 1 and 2 trillion US dollars [4]. Each medical discipline is characterized by its own unique set of AEs, reflecting the specific risks and complications associated with its procedures and patient populations.

In obstetrics, AEs have significant implications, affecting not only the expectant mother, newborn, and future family but also the treating healthcare team, which must cope with the ramifications of treatment errors and potential medicolegal consequences [5]. The absence of a uniform or specialty-specific definition of AEs makes it challenging to compare them quantitatively and qualitatively. This lack of standardization leads to difficulties in accurately assessing and evaluating AEs across different medical specialties or healthcare settings. One definition is provided in the Institute of Medicine (IOM) report 'To Err Is Human: Building a Safer Health System' as 'the failure of a planned action to be completed as intended or the use of a wrong plan to achieve an aim' [6]. To evaluate and measure AE rates, structured methods such as the Harvard Medical Practice Study (HMPS) and the Global Trigger Tool (GTT) could be used for structured chart reviews. Findings show that medication errors are the most common AEs, with an estimated proportion of 28 % being preventable [2,7,8]. In obstetrics, the prevalence of AEs is estimated to be 1–4 %, with half of them classified as preventable [[9], [10], [11]]. Inadequate teamwork and poor communication among obstetric healthcare professionals are often identified as key contributing factors [12].

Besides its impact on patient safety, communication training could be vital for women with social risk factors in maternity care, as it addresses stigma, discrimination, and resulting disengagement from services. By promoting woman-centered care and improving trust in healthcare professionals, such training can lead to better uptake of support, reliance on trusted advice, and active participation in their care. It is also important to acknowledge the role of the partner in maternity care both in providing support to the woman giving birth and as part of the family receiving care. This illustrates the complexity of communication and the need for explicit partner involvement for birth outcomes and family adjustment [13,14]. Enhancing communication and trust through a more family-centered approach is essential for improving outcomes in maternity care [15].

Critical error analysis reveals that many staff members in maternity care do not receive communication training in crucial areas identified as leading causes of harm and fatalities in maternity care. A lack of resources and financial support was identified as a significant barrier to providing such essential training [16,17].

In light of this fact, and in addition to the systematic recording of medical treatment errors and their root cause analysis [18,19], the reduction through targeted patient training intervention is a crucial approach in clinical risk management [20]. Recognizing that AEs are also influenced by human factors [21] and that pAEs can be attributed to *inadequate communication* in up to 70 % of cases [22], critical communication needs improvement [23].

In a previous study, a classification of AEs and pAEs in obstetrics was established [24]. Subsequently, in the context of a case-control study, a significant reduction of pAEs was achieved following focused communication training for the healthcare professionals in obstetrics [25]. These empirical findings not only underscore the intrinsic value of effective communication training but also substantiate its efficacy in enhancing patient safety.

In a second phase of the above-mentioned study, expectant mothers and their partners were also trained in targeted communication for childbirth. While an improvement in the childbirth experience was observed, no significant effect on self-reported AEs was demonstrated [26]. However, in the current era of healthcare reform, the focus is not only on improving care quality subjectively but also on objective cost containment. Economic evaluations play a vital role in translating research into evidence-based practices and guiding policy decisions [27]. Beyond evaluating the effectiveness of targeted communication training, research needs to establish the cost effectiveness of implementing such training protocols [28]. In obstetrics, there remains a dearth of viable analyses on this matter, as many cost analyses in medicine primarily focusing on medical screening methods or specific treatment programs.

Cost effectiveness refers to the economic evaluation of an intervention in relation to its outcomes, assessing whether the benefits justify the costs incurred [28]. In healthcare, cost effectiveness is often used to compare different treatment strategies to determine which provides the best value for money [27].

Cost effectiveness analysis is typically used to assess the relative costs and outcomes of different interventions or a new intervention in comparison to usual care, typically expressed as a cost per unit of health benefit (e.g., cost per birthing procedure) [35]. In this context, a Bayesian generalized linear model can be used to estimate the impact of a patient training intervention on healthcare costs, after controlling pre-existing maternal and fetal risk factors. This approach allows for an evaluation of the effect of targeted communication training in obstetrics, accounting for uncertainty and individual variability in cost outcomes.

Therefore, the present analysis of an experimental efficacy case-control study performed a cost effectiveness analysis with a Bayesian generalized linear model to estimate the impact of the patient training intervention on healthcare costs, after controlling for pre-existing maternal and fetal risk factors to demonstrate the effect of targeted communication training in obstetrics. As women in the intervention group reported better communication and birth experiences, both constructs related to patient safety, the intervention might be cost effective.

## 2 Methods

The analyzed data is derived from a study conducted as part of a larger research project (Clinical Trials gov. Identifier: NCT03855735). The study protocol has been published elsewhere [29]. The aim of the study was to improve effective communication and thus increase patient safety in obstetrics. The project was funded by the Innovation Fund (project no. 01VSF18023) of the Federal Joint Committee (G-BA). The cost data analyzed in this manuscript were collected following a randomized-controlled trial testing communication training for pregnant women and their partners, compared to care-as-usual, which has been published before [26].

### 2.1 Recruitment

The research was conducted at Perinatal Center Level I in a university hospital. The recruitment process was managed by the hospital's internal project staff, and eligible participants included expectant mothers and their partners proficient in German, aged 17 years or older, and planning to give birth at the study hospital. These expectant mothers were largely cared for by the same providers who had received focused communication training in an earlier phase of the project, after which a significant reduction in pAEs was achieved [24,25].

Recruitment efforts involved various channels such as distributing flyers, posters, and registration forms within the hospital, informing resident gynecologists, midwives, and pregnancy counselling services, as well as promoting the project on hospital websites, social media, and through press releases. Participants received study information and baseline questionnaires via email and provided informed consent through the online questionnaire. After completing the questionnaires, participants were informed of their group assignment (training-intervention or control). *N* = 196 women who were interested in the study provided questionnaire data at T1. Of these, 160 (81.6 %) were between 30 and 34 years old. Most were pregnant with their first (122, 62.2 %) child, came from a German background (172, 87.8 %), and had a university degree (136, 69.4 %). The majority were married or in a stable relationship (186, 94.9 %). The mean week of pregnancy was 33.85 (*SD* = 3.68). Overall, 76 women and their partners were randomly allocated to the training arm, while 88 were allocated to the control arm and gave birth at the hospital, allowing their birth data to be analyzed in the cost analysis. A previous evaluation showed that women in the training arm had higher self-reported communication behavior, better coping planning and a higher perceived quality of giving birth in intention-to-treat analyses after the training compared to the control arm [26].

### 2.2 Data collection

To evaluate the cost effectiveness of the patient training intervention, the objective obstetric data from the women participating in the study were collected and evaluated. Participants were assigned to either the training or control arm. Each birth was noted with a Diagnosis Related Groups (DRG) classification, internal treatment cost, reimbursement by health insurance, as well as patient characteristics, co-morbidities, and risk factors. A nurse compiled the data into a format suitable for quantitative analysis and to derive preliminary estimates of the cost effectiveness of communication training.

### 2.3 Patient training

A company, in close cooperation with the research team, developed and conducted effective communication training for expectant mothers. This training was adapted from a previous training for obstetric healthcare workers now tailored to pregnant women and their partners’ needs. The training aimed to raise awareness of patient safety risks related to communication and motivate behavior change toward safe communication. The training included empathy-building exercises, practicing communication competencies, and developing individual communication strategies, as outlined in detail by Derksen et al. [30]. The *Health Action Process Approach* (HAPA) framework guided the training, focusing on behavioral determinants like raising awareness about patient safety risks due to inadequate communication and motivating behavior change. It also enhanced self-efficacy and communication planning. Behavior Change Techniques were chosen accordingly.

The training was conducted online due to safety regulations during the corona-pandemic, as the training was delivered between June 2020 and October 2021. The training began with participants sharing their ideal childbirth expectations and prior experiences, aiding goal setting and commitment. They engaged in perspective-taking, fostering mutual understanding and social support. Communication skills were practiced, including "speaking up" and "closed-loop communication" techniques. Participants developed tailored communication strategies. The training lasted about 2.5 h, with a 5–10 min break plus a 5-min active component with physical exercises [30].

### 2.4 Data processing

The DRG classifications were used to classify births as either complicated or non-complicated cases. Complications primarily include unplanned healthcare services such as secondary caesarean sections, preterm births, or vaginal births with postpartum complications. In the appendices Table A.1 presents a list of DRGs assigned to each group. The impact of the patient training intervention was evaluated for each group separately to evaluate the effect of the patient training intervention in both scenarios.

### 2.5 Data analysis

Study participants were categorized into *two groups*: those who experienced complications during childbirth and those who did not (see Table 1, Table 2). For each group, a *Bayesian generalized linear model* was used to estimate the impact of patient training on healthcare costs, after controlling for pre-existing maternal and fetal risk factors. The Bayesian model was selected because prior evidence on cost savings was available from the same clinic regarding a communication training intervention. When prior data are available, Bayesian models are preferred because they leverage more data to derive intervention effect estimates relative to Frequentist models (i.e., utilizing both observed data and prior beliefs in estimating the effect), providing a higher level of confidence in the final results.

**Table 1:** Parameter estimated and the credible intervals of variables predicting the percentage change in costs for *complicated* births.

Estimate	Mean	Standard deviation	Credible interval
First time birth	−20 %	10 %	{-40 %, −10 %}
After-hours delivery	−20 %	10 %	{-30 %, −10 %}
Other maternal risk	20 %	10 %	{0 %, 40 %}
Fetal risk	30 %	10 %	{10 %, 40 %}
Patient training intervention	−30 %	10 %	{-50 %, −20 %}

**Table 2:** Parameter estimated and the credible intervals of variables predicting the percentage change in costs for *uncomplicated* births.

Estimate	Mean	Standard deviation	Credible interval
First time birth	30 %	10 %	{10 %, 40 %}
After-hours delivery	−30 %	10 %	{-40 %, −10 %}
Other maternal risk	20 %	10 %	{10 %, 30 %}
Fetal risk	−10 %	10 %	{-20 %, 10 %}
Patient training intervention	−10 %	10 %	{-20 %, 10 %}

A description of the independent and dependent variables used in the model is presented in Table A.2 in the appendices. Data processing and analyses were performed using *R* Statistical Software (v4.3.1; R Core Team 2023).

## 3 Results

### 3.1 Cost effectiveness analysis

Descriptive statistics demonstrate lower costs associated with the intervention group. For patients with complications, the average cost of care for those who received the intervention and those in the control group was 3053 Euros and 4523 Euros, respectively. For patients without complications, the average cost of care for those who received the intervention and those in the control group was 2168 and 2418 Euros, respectively. Further analyses comparing the groups and estimating the effect of the intervention are presented below.

In the cost effectiveness analysis, a significant training effect was found for patients with complications (*M* = −30, *SD* = 10, 95 % CI [−50, −20]); the model estimated that patient training reduced average patient costs by 30 %. There was no significant training effect for those without complications (*M* = −10, *SD* = 8, 95 % CI [−20, 10]), which is likely due to the small variation in the costs for this patient group and the lower costs compared to the group with complications. The effect of environmental and individual risk factors on the costs of care was controlled for in both models.

### 3.2 Patients with complications

**Fig. 1:**
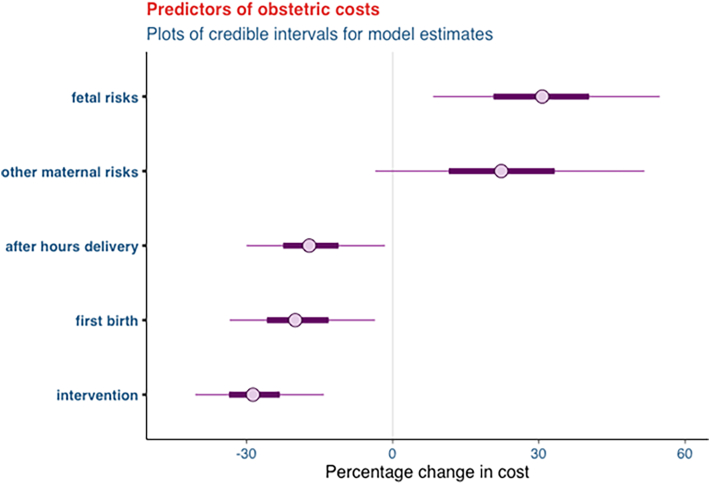
Predictors of obstetric costs for *complicated* births.

Fig. 1 demonstrate the impact of the patient training intervention, after controlling for pre-existing maternal and fetal risk factors for women with *complicated* births. Pre-existing maternal risk factors include age, parity, pre-existing conditions, pregnancy-associated diseases, drug abuse, and allergies. Pre-existing fetal risk factors include growth restriction, large for gestational age, and the fetal position at birth.

The credible intervals highlight that hospitals could save between 20 % and 50 % per patient for complicated births if the patient training intervention is implemented (see Table 1). Moreover, a first birth or after-hours delivery was associated with a lower cost of care, while the presence of fetal risks was associated with a higher cost.

### 3.3 Patients without complications

**Fig. 2:**
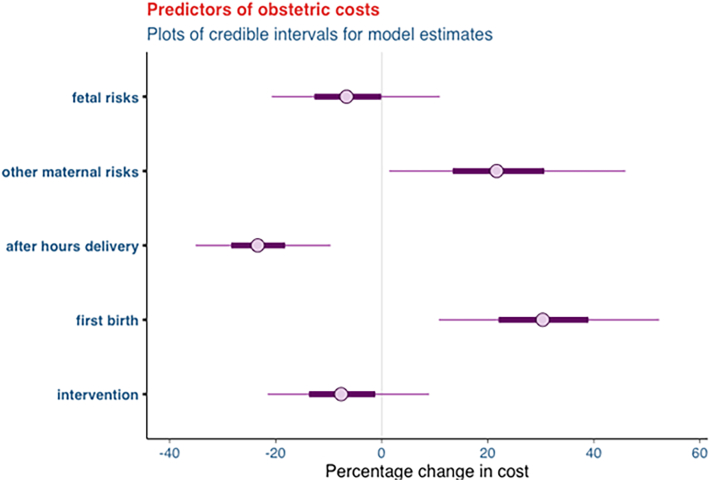
Predictors of obstetric costs for *uncomplicated* births.

Fig. 2 only show the descriptive effect of the patient training intervention after controlling for pre-existing maternal and fetal risk factors for women with *uncomplicated* births.

Significant effects were due to after-hours deliveries, which were associated with a lower cost of care, first time birth and other maternal risk factors which were associated with a greater cost of care (see Table 2).

## 4 Discussion

For Germany in the year 2022, based on available and extrapolated values, a further increase in healthcare expenditures was estimated to reach 498.1 billion Euros. This would represent an additional 24 billion Euros or 5.1 % more than in 2021 [31]. Due to rising healthcare costs, various approaches can be used to curb the trend in healthcare expenditures: enhancing the efficiency of healthcare delivery, increasing financial incentives for patients to limit the utilization of medical services, strengthening administrative controls on the utilization of these services, or limiting the resources available to the healthcare system [32] by means of patient training. However, it is essential not to jeopardize medical care and patient safety with these approaches. A cost-benefit analysis (i.e. so-called trial-based cost effectiveness evaluations), alongside randomized clinical trials, appears crucial in this context [33]. Healthcare costs and awareness of economic factors are rising across all areas of medicine. Obstetrics, in particular, significantly contributes to per-patient costs in the healthcare system. Thus, the current study performed a trial-based cost effectiveness evaluation in obstetrics, demonstrating the efficacy of patient training aimed at effective communication with objective data. We found clear support for the effect of communication training on costs.

In a previous study with questionnaire data, we analyzed potential effects perceived by the patients. We found that action planning, coping planning, coping self-efficacy, and perceived quality of birth increased more in the intervention group than the passive control group [26]. Although this effect was not observed for communication behavior itself, it is likely that these specific mechanisms, based on the intervention content rather than random effects such as attention, drove the positive effects found in the cost effectiveness analysis.

Our findings are in line with those of a meta-analysis that included 22 obstetric cost-benefit analyses. Different treatment methods, obstetric prenatal care management, therapies for prematurity, and induction of labor regimes were compared. The results primarily examined obstetric outcome parameters such as caesarean section rates, obstetric complications, and success rates in therapy. Overall, the quality of the studies was not rated very high [33]. None of these studies examined the effect of improved communication on healthcare costs in obstetrics. However, our study overcame these problems and, thus, comparability of the data is not possible. Our study can be seen as a role model for future research. However, also our study had some limitations, such as the lack of a baseline measure of healthcare costs, the comparison to a placebo intervention, and the inability to investigate what exactly determined the effectiveness of the intervention. Researching this was ethically and practically not possible to realize in the current study, but the data still speaks for the effects of the intervention on patients’ communication and patient safety.

Recently, a study protocol for improved communication in nursing was published and will also assess a cost–benefit analysis as a secondary endpoint [34]. Fundamentally, every cost effectiveness analysis should include two reference case analyses: one from the healthcare sector's perspective and one from the societal perspective [35]. These approaches can also be applied to our analysis, which has only evaluated healthcare costs so far. Nevertheless, the implementation of professionally guided communication training for expectant mothers and their partners has demonstrated a measurable impact on satisfaction with the childbirth experience, illustrating a potentially positive influence on the course of labor [26,36].

In the cost analysis, a measurable effect was particularly observed in births with complications, leading to a meaningful reduction in costs. This is in line with findings from the questionnaire analysis, showing that self-efficacy and planning of communication behavior in difficult situations (coping with challenges such as birth complications) improved. On the other hand, the influence of pre-existing risk factors is challenging to establish, as they are not easily controllable. Obstetric costs are higher in cases of fetal risks, while they are lower in primiparous women, who are often younger and without severe obstetric risk factors. However, it was difficult to estimate the effect of the patient training intervention on women with no complications. This was probably due to the small variation in costs for women with no complications.

From a societal perspective, the implementation of communication training does not lead to the rationalization of medical standards and, thus, does not endanger patient safety. Moreover, an improved, effective and empathic communication increases patient satisfaction and patient safety [37,38]. In the observed cohort in this study, the patient training and communication intervention increased the perceived quality of birth and self-efficacy in dealing with difficult situations [26]. Additionally, person-centered communication improved the self-efficacy of healthcare workers [39]. This is an important aspect when considering cost effectiveness in obstetrics.

Restrictive healthcare management, such as reductions in preventive obstetric care or an increased focus on outpatient management, can lead to a deterioration of obstetric outcomes. However, this was not the case in the current patient training intervention. Nevertheless, the patient training intervention for pregnant women attracted highly educated, native-speaking individuals from an ethnic majority background with a previous awareness of the crucial role of communication [26].

Adverse outcomes may also be associated with specific risk factors for fetal and maternal complications. Often, these originate from so-called structural risk factors, such as low levels of education and income, migrant status, and living in disadvantaged areas [40].

Patient training interventions that do not specifically focus on the needs of these at-risk groups and ethnic minority groups have been shown to widen existing equity gaps [41], which might have also been the case in the patient training intervention introduced in this study. Thus, future implementation efforts should identify and explicitly target groups facing inequalities in maternity care to achieve equity. In this way, future communication interventions can specifically improve obstetric care for women who are at higher risk for complications. This is especially imperative for risk management: In the case of complications, patient safety and communication training have shown significant effects. While there was no harm to patients without risk factors, the benefit was not significant and may not have outweighed the costs of the training. However, for patients with risk factors, such training appears much less expensive compared to the lower costs of 4523 Euros versus 3053 Euros.

Therefore, targeted communication interventions could foster fairness by ensuring all women, especially the most vulnerable, have access to high-quality maternal health services, utilizing effective communication to pinpoint and fulfill their requirements. Prioritizing effective communication training could elevate the standard of patient care and bolster the robustness and resilience of health systems through improvements in the health workforce and healthcare facilities [42].

Our analysis underscores the essential role of respectful, informed, and woman-centered communication in the delivery of effective and equitable maternal healthcare services. It highlights its importance in enhancing patient satisfaction and outcomes, as well as in the broader context, improving global maternal health standards. This is in line with current literature which emphasizes the urgency of a global approach to quality and equitable maternal health, supporting the implementation of respectful, evidence-based care for all [43].

## 5 Conclusion

The pressure on the cost effectiveness of healthcare is increasing and patient training intervention can buffer the need for such healthcare services i.e. the costs due to elevated healthcare costs. The effects of interventions must be transparently measurable for implementation. Without this evidence, new prevention methods, in particular, are not introduced. In general, it is emphasized that active and self-determined participation in childbirth can have a significant impact on the subsequent mother-child relationship. A patient-centered care approach, along with guidance on communication for making interactive decisions, is essential [44]. Therefore, the introduction of patient training intervention, such as the evaluated communication training for expectant parents, bridges the gap between economic efficiency and future-oriented interventions in obstetrics. This approach not only improves the cost effectiveness of care but also ensures equitable access to quality maternal services, emphasizing the importance of equality in achieving optimal outcomes for all mothers.

## Data availability

All data is presented in the article.

## Funding statement

This work was supported by the Innovation Fund (project no. 01VSF18023) of the Federal Joint Committee (G-BA).

This program of work was partly funded by Health CASCADE. HealthCASCADE is a Marie Sklodowska-Curie Innovative Training Network funded by the European Union's Horizon 2020 research and innovation program under Marie Sklodowska-Curie grant agreement number 956501.

Parts of this manuscript were conducted as part of the Rehabilitation Research Priority Professorship in cooperation with the German Pension Insurance Association, funded by the BMBF and the BWFGB as part of the federal-state program “FH-Personal” (go-2-prof:in project at HAW Hamburg).

## Authors‘ contribution

The study was conceived and designed by SL and VAK. SL , VAK and HB were responsible for data collection and analysis and drafting of the manuscript. SM, DC, SC and RF made critical revisions to the manuscript for important intellectual content. The final version of the manuscript was read and approved for submission by all authors.

## Declaration of competing interest

All authors declare no conflict of interest.

## References

[1] Miziara I.D., Miziara C.S.M.G. (2022). Medical errors, medical negligence and defensive medicine: a narrative review. Clinics.

[2] Oyebode F. (2013). Clinical errors and medical negligence. Med. Princ. Pract..

[3] Vikan M., Haugen A.S., Bjørnnes A.K., Valeberg B.T., Deilkås E.C.T., Danielsen S.O. (2023). The association between patient safety culture and adverse events – a scoping review. BMC Health Serv. Res..

[4] Slawomirski L., Auraaen A., Klazinga N.S. (2017).

[5] Schrappe M., Müller H., Hecker R. (2020). [Patient safety: current problems and challenges]. Internist.

[6] Kohn L.T., Corrigan J., Donaldson M.S. (2000). To Err Is Human: Building a Safer Health System.

[7] Bates D.W., Boyle D.L., Vander Vliet M.B., Schneider J., Leape L. (1995). Relationship between medication errors and adverse drug events. J. Gen. Intern. Med..

[8] Agbar F., Zhang S., Wu Y., Mustafa M. (2023). Effect of patient safety education interventions on patient safety culture of health care professionals: systematic review and meta-analysis. Nurse Educ. Pract..

[9] Aibar L., Rabanaque M.J., Aibar C., Aranaz J.M., Mozas J. (2015). Patient safety and adverse events related with obstetric care. Arch. Gynecol. Obstet..

[10] Forster A.J., Fung I., Caughey S., Oppenheimer L., Beach C., Shojania K.G. (2006). Adverse events detected by clinical surveillance on an obstetric service. Obstet. Gynecol..

[11] Pettker C.M., Thung S.F., Norwitz E.R., Buhimschi C.S., Raab C.A., Copel J.A. (2009). Impact of a comprehensive patient safety strategy on obstetric adverse events. Am. J. Obstet. Gynecol..

[12] Stierman E.K., Kramer B., Bower K.M., Creanga A.A. (2024). A call to address teamwork and patient safety culture in hospital maternity units: findings from a survey of maternal healthcare professionals in Maryland. Am. J. Obstet. Gynecol..

[13] Schmitt N., Striebich S., Meyer G., Berg A., Ayerle G.M. (2022). The partner's experiences of childbirth in countries with a highly developed clinical setting: a scoping review. BMC Pregnancy Childbirth.

[14] Sudhinaraset M., Landrian A., Golub G.M., Cotter S.Y., Afulani P.A. (2021). Person-centered maternity care and postnatal health: associations with maternal and newborn health outcomes. AJOG Glob Rep.

[15] Rayment-Jones H., Harris J., Harden A., Turienzo C.F., Sandall J. (2023). Project20: maternity care mechanisms that improve (or exacerbate) health inequalities. A realist evaluation. Women Birth.

[16] Lambert MD Georgia (2024).

[17] NHS Resolution - Annual Report and Accounts 2022/23 n.d.

[18] White A.A., Pichert J.W., Bledsoe S.H., Irwin C., Entman S.S. (2005). Cause and effect analysis of closed claims in obstetrics and gynecology. Obstet. Gynecol..

[19] Pettker C.M. (2017). Systematic approaches to adverse events in obstetrics, Part I: event identification and classification. Semin. Perinatol..

[20] Leape L.L., Brennan T.A., Laird N., Lawthers A.G., Localio A.R., Barnes B.A. (1991). The nature of adverse events in hospitalized patients. Results of the Harvard Medical Practice Study II. N. Engl. J. Med..

[21] Reason J. (1995). Understanding adverse events: human factors. Qual. Health Care.

[22] Leonard M., Graham S., Bonacum D. (2004). The human factor: the critical importance of effective teamwork and communication in providing safe care. Qual. Saf. Health Care.

[23] Lippke S., Derksen C., Keller F.M., Kötting L., Schmiedhofer M., Welp A. (2021). Effectiveness of communication interventions in obstetrics-A systematic review. Int J Environ Res Public Health.

[24] Hüner B., Derksen C., Schmiedhofer M., Lippke S., Janni W., Scholz C. (2022). Preventable adverse events in obstetrics-systemic assessment of their incidence and linked risk factors. Healthcare (Basel).

[25] Hüner B., Derksen C., Schmiedhofer M., Lippke S., Riedmüller S., Janni W. (2023). Reducing preventable adverse events in obstetrics by improving interprofessional communication skills – results of an intervention study. BMC Pregnancy Childbirth.

[26] Derksen C., Dietl J.E., Haeussler F.E., Steinherr Zazo M., Schmiedhofer M., Lippke S. (2023). Behavior change training for pregnant women's communication during birth: a randomized controlled trial. Appl Psychol Health Well Being.

[27] Rodriguez M.I., Caughey A.B. (2013). Cost-effectiveness analyses and their role in improving healthcare strategies. Curr. Opin. Obstet. Gynecol..

[28] Hutchinson P., Wheeler J. (2006). The cost-effectiveness of health communication programs: what do we know?. J. Health Commun..

[29] Lippke S., Wienert J., Keller F.M., Derksen C., Welp A., Kötting L. (2019). Communication and patient safety in gynecology and obstetrics - study protocol of an intervention study. BMC Health Serv. Res..

[30] Derksen C., Kötting L., Keller F.M., Schmiedhofer M., Lippke S. (2022). Psychological intervention to improve communication and patient safety in obstetrics: examination of the health action process approach. Front. Psychol..

[31] Destatis. Gesundheitsausgaben. Statistisches Bundesamt n.d. https://www.destatis.de/DE/Presse/Pressemitteilungen/2023/04/PD23_136_236.html (accessed October 27, 2023).

[32] Ginsburg P.B. (2004). Controlling health care costs. N. Engl. J. Med..

[33] El Alili M., van Dongen J.M., Huirne J.A.F., van Tulder M.W., Bosmans J.E. (2017). Reporting and analysis of trial-based cost-effectiveness evaluations in obstetrics and gynaecology. Pharmacoeconomics.

[34] Liu M., Whittam S., Thornton A., Goncharov L., Slade D., McElduff B. (2023). The ACCELERATE Plus (assessment and communication excellence for safe patient outcomes) Trial Protocol: a stepped-wedge cluster randomised trial, cost-benefit analysis, and process evaluation. BMC Nurs..

[35] Sanders G.D., Neumann P.J., Basu A., Brock D.W., Feeny D., Krahn M. (2016). Recommendations for conduct, methodological practices, and reporting of cost-effectiveness analyses: second panel on cost-effectiveness in health and medicine. JAMA.

[36] Schmiedhofer M., Derksen C., Dietl J.E., Haeussler F., Strametz R., Huener B. (2022). The impact of a communication training on the birth experience: qualitative interviews with mothers after giving birth at obstetric university departments in Germany. Int J Environ Res Public Health.

[37] Byrne M., Campos C., Daly S., Lok B., Miles A. (2024). The current state of empathy, compassion and person-centred communication training in healthcare: an umbrella review. Patient Educ. Counsel..

[38] Oliveira V.C., Ferreira M.L., Pinto R.Z., Filho R.F., Refshauge K., Ferreira P.H. (2015). Effectiveness of training clinicians' communication skills on patients' clinical outcomes: a systematic review. J. Manipulative Physiol. Therapeut..

[39] Wolderslund M., Kofoed P.-E., Ammentorp J. (2021). The effectiveness of a person-centred communication skills training programme for the health care professionals of a large hospital in Denmark. Patient Educ. Counsel..

[40] de Graaf J.P., Steegers E.A.P., Bonsel G.J. (2013). Inequalities in perinatal and maternal health. Curr. Opin. Obstet. Gynecol..

[41] Yuan B., Målqvist M., Trygg N., Qian X., Ng N., Thomsen S. (2014). What interventions are effective on reducing inequalities in maternal and child health in low- and middle-income settings? A systematic review. BMC Public Health.

[42] Koblinsky M., Moyer C.A., Calvert C., Campbell J., Campbell O.M.R., Feigl A.B. (2016). Quality maternity care for every woman, everywhere: a call to action. Lancet.

[43] Miller S., Abalos E., Chamillard M., Ciapponi A., Colaci D., Comandé D. (2016). Beyond too little, too late and too much, too soon: a pathway towards evidence-based, respectful maternity care worldwide. Lancet.

[44] Altgeld T., Kuhn A. (2017). Nationales Gesundheitsziel - Gesundheit rund um die Geburt. National Health Goal - Health Around Birth.

