# Supplementary material for: Cost effective analysis after patient communication training in obstetrics - Evaluating economic efficiency
Source: Public Health Pract (Oxf). 2025 May 13;9:100618. doi: 10.1016/j.puhip.2025.100618 (PMC12148821; doi:10.1016/j.puhip.2025.100618)
Supplement: Multimedia component 1 [file mmc1.docx]

Table A.1: List of obstetric DRG classifications - Categorization into *uncomplicated* and *complicated*

| DRG | Description | Status |
| --- | --- | --- |
| O01F | Primary caesarean section without complex diagnosis, duration of pregnancy > 33 completed weeks (Weeks of pregnancy, WOG). | *Uncompli­cated* |
| O60D | Vaginal delivery without complicating diagnosis, duration of pregnancy > 33 completed WOG. |  |
| O65B | Other prenatal inpatient admission without extremely severe or severe complicating constellation, without complex diagnosis, without complicating procedure, more than one day of occupancy. |  |
| O01A | Secondary caesarean section with multiple complicating diagnoses up to 25 completed WOG, or with intrauterine therapy, or complicating constellation, or multiple pregnancy. | *Compli­cated* |
| O01B | Caesarean section, pregnancy up to 25 completed WOG, with more complicating diagnosis, with intrauterine therapy or complicating constellation or multiple pregnancy or up to 33 completed WOG or with complicated diagnosis, with or without complicated diagnosis. Diagnosis with medical procedure in case of caesarean section or extreme severe complicating constellation. |  |
| O01C | Caesarean section with multiple complications. Duration of pregnancy 26 to 33 completed WOG, without certain complicating factors or with complicating factors. Factors or with complicating diagnosis up to 25 completed WOG or with tamponade of a hemorrhage or thromboembolism in gestational period with operating room process, without severe complicating constellation. |  |
| O01D | Secondary caesarean section with multiple complications. Diagnosis, duration of pregnancy > 33 completed WOG, or intrauterine therapy, no multiple pregnancy or up to 33 completed WOG or with complicated diagnosis, with or without complicated diagnosis. Diagnosis without severe complicating constellation. |  |
| O01E | Primary caesarean section without extremely severe complicating constellation, with complicating or complex diagnosis or duration of pregnancy up to 33 completed WOG or secondary caesarean section, without complex diagnosis, duration of pregnancy > 33 completed WOG. |  |
| O01G | Primary caesarean section with complicating diagnosis, duration of pregnancy more than 33 completed WOG, without complex diagnosis. |  |
| O02A | Vaginal delivery with complicating operating room procedure, duration of pregnancy up to 33 completed WOG or with intrauterine therapy or complicating constellation or specific medical procedure or complicating diagnosis or with extremely severe complicating constellation. |  |
| O02B | Vaginal delivery with complicating operating room procedure, duration of pregnancy more than 33 completed WOG, without intrauterine therapy, without complicating constellation, without specific medical procedure, without complicating diagnosis, without extremely severe complicating constellation. |  |
| O04A | Inpatient admission after delivery or miscarriage with operating room procedure or specific procedure on the mamma with complex procedure. |  |
| O04B | Inpatient admission after delivery or miscarriage with operating room procedure or specific procedure on the mamma, without complex procedure. |  |
| O04C | Inpatient admission after delivery with minor surgery to the uterus, vagina, perianal region, abdominal wall or abortion with dilation and curettage, aspiration curettage or hysterotomy, or certain amniotic puncture. |  |
| O60A | Vaginal delivery with multiple complicating diagnoses, at least one severe, duration of pregnancy up to 33 completed WOG or with complicating constellation. |  |
| O60B | Vaginal delivery with more complicating diagnosis, at least one difficulty > 33 completed WOG without complicating constellation or tamponade of hemorrhage or thromboembolism during the gestational period without operating room process or severe or moderately severe complications. Operating room process or severe or moderately severe complicated constellation. Diagnosis up to 33 completed WOG. |  |
| O60C | Vaginal delivery with severe or moderately severe complicating diagnosis or duration of pregnancy up to 33 completed weeks. |  |
| O61Z | Inpatient admission after delivery or miscarriage without operating room procedure, without specific mammary intervention. |  |
| O65A | Other prenatal inpatient admission with extremely severe or severe complicating constellation or complex diagnosis or complicating procedure or one day of occupancy. |  |

Table A.2: Variables used in the predictive models

| Variable group | Variable name | Description |
| --- | --- | --- |
| IV | After hours delivery | A binary variable indicating if baby was delivered after hours (1) or not (0). |
|  | First birth | A binary variable indicating if it was the first birth (1) or not (0). |
|  | Intervention | A binary variable indicating if participant was in training (1) or control (0) arm. |
|  | Fetal risks | A binary variable indicating if there was elevated risk related to the fetus (1) or not (0). |
|  | Other maternal risk factors | A binary variable indicating if at least one risk factor outside of those already noted in the existing IVs was recorded for the individual (0) or not (1). |
| DV | Costs per patient | An estimate (in Euros) of the cost of delivering the baby and providing care to the patient. |


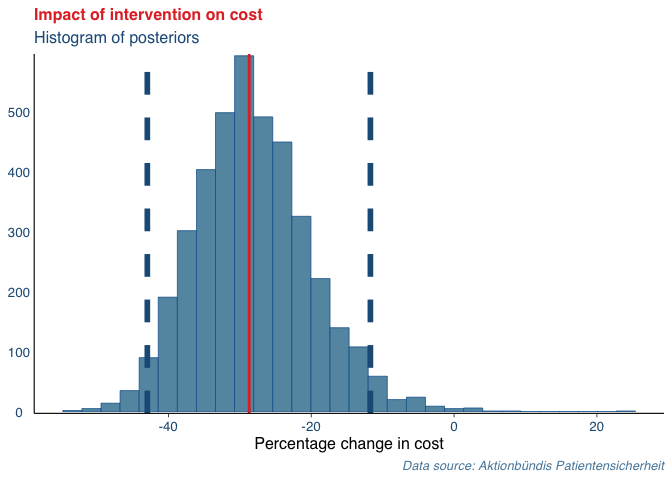


Figure A.1: Histogram presenting the distribution of the estimates of the training effect on cost for *complicated* births


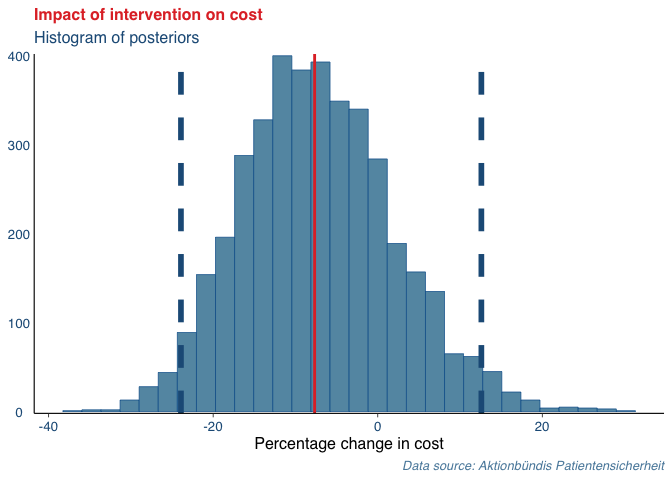


Figure A.2: Histogram presenting the distribution of the estimates of the training effect on cost for *uncomplicated* births
